# Non-falciparum species and submicroscopic infections in three epidemiological malaria facets in Cameroon

**DOI:** 10.1186/s12879-022-07901-6

**Published:** 2022-12-02

**Authors:** Loick Pradel Kojom Foko, Joseph Hawadak, Francine Dorgelesse Kouemo Motse, Carole Else Eboumbou Moukoko, Lugarde Kamgain Mawabo, Veena Pande, Vineeta Singh

**Affiliations:** 1 ICMR-National Institute of Malaria Research, Dwarka, New-Delhi 110077 India; 2 Department of Biotechnology, Kumaun University, Bhimtal, Uttarakhand 263001 India; 3 Department of Medical Laboratory Sciences, Faculty of Health Sciences, University of Buea, 63, Buea, Cameroon; 4 Department of Biological Sciences, Faculty of Medicine and Pharmaceutical Sciences, The University of Douala, 24157 Douala, Cameroon; 5 Malaria Research Unit, Centre Pasteur Cameroon, 1274 Yaoundé, Cameroon; 6 Laboratory of Parasitology, Mycology and Virology, Postgraduate Training Unit for Health Sciences, Postgraduate School for Pure and Applied Sciences, The University of Douala, 24157 Douala, Cameroon; 7 Laboratoire Sainte Therese, Douala, Cameroon

**Keywords:** Malaria, Non-falciparum species, Submicroscopic infections, Cameroon

## Abstract

**Background:**

There are growing reports on the prevalence of non-falciparum species and submicroscopic infections in sub-Saharan African countries but little information is available from Cameroon.

**Methods:**

A hospital-based cross-sectional study was carried out in four towns (Douala, Maroua, Mayo-Oulo, and Pette) from three malaria epidemiological strata (Forest, Sahelian, and Soudanian) of Cameroon. Malaria parasites were detected by Giemsa light microscopy and polymerase chain reaction (PCR) assay. Non-falciparum isolates were characterized and their *18S* gene sequences were BLASTed for confirmatory diagnosis.

**Results:**

PCR assay detected malaria parasites in 82.4% (98/119) patients, among them 12.2% (12/98) were asymptomatic cases. Three *Plasmodium* species viz. *P.* *falciparum*, *P.* *ovale* *curtisi* and *P.* *vivax*, and two co-infection types (*P.* *falciparum* + *P.* *vivax* and *P.* *falciparum* + *P.* *ovale* *curtisi*) were found. The remaining infections were mono–infections with either *P.* *falciparum* or *P.* *ovale* *curtisi*. All non–falciparum infections were symptomatic and microscopic. The overall proportion of submicroscopic infections was 11.8% (14/119). Most asymptomatic and submicroscopic infection cases were self-medicated with antimalarial drugs and/or medicinal plants. On analysis, *P.* *ovale* *curtisi* sequences were found to be phylogenetically closer to sequences from India while *P.* *vivax* isolates appeared closer to those from Nigeria, India, and Cameroon. No G6PD-d case was found among non-falciparum infections.

**Conclusions:**

This study confirms our previous work on circulation of *P.* *vivax* and *P.* *ovale* *curtisi* and the absence of *P.* *knowlesi* in Cameroon. More studies are needed to address non-falciparum malaria along with submicroscopic infections for effective malaria management and control in Cameroon.

**Supplementary Information:**

The online version contains supplementary material available at 10.1186/s12879-022-07901-6.

## Background

Malaria is a disease transmitted to humans through the bite of female *Anopheles* mosquitoes infected with *Plasmodium* parasites [1]. Till date, five *Plasmodium* species can cause malaria in humans such as *P.* *falciparum*, *P.* *vivax*, *P.* *ovale* *spp*, *P.* *malariae*, and *P.* *knowlesi* [1, 2]. The World Health Organization (WHO) reported that malaria was responsible for 241 million disease cases and 627,000 deaths worldwide in 2020 [3]. Sub-Saharan Africa (sSA) is known to be the most affected region of the world suffering from malaria disease with over 90% of the figures estimated by the WHO. The most vulnerable groups infected with malaria parasites among the population remain children under five years and pregnant women [3].

*Plasmodium* *falciparum* is the main species involved in malaria burden globally [4]. A large number of control methods such as artemisinin-based combination therapies (ACT) and long-lasting insecticide-treated nets have been developed and/or scaled up to control *P.* *falciparum* in endemic areas [4]. As a result, a substantial decrease in *P.* *falciparum* malaria burden has been observed the last decade [5]. Interestingly, the current epidemiology of *P.* *falciparum* along with that of non-falciparum species has probably changed due to these control methods. For instance, the recent change in *P.* *knowlesi* epidemiology in some Southeast Asian (SEA) regions like Malaysia is a typical example [6, 7]. To achieve WHO malaria control and elimination objectives by 2030, non-falciparum species should also be taken into consideration [5]. In view of this, it is of utmost importance to adequately determine the epidemiology of *Plasmodium* species using reliable and sensitive diagnostic tools.

Light microscopy (LM) and immunochromatographic rapid diagnostic tests (RDTs) have been the mainstay tools used for diagnosis of malaria [5], but they are not sufficiently reliable for diagnosis of non-falciparum species [8, 9]. The main reasons are attributed to the difficulties faced in differentiating the species due to their morphological similarities (i.e., *P.* *vivax* and *P.* *ovale* *spp*, or *P.* *falciparum* and *P.* *knowlesi*) as well as low parasite densities observed in non-falciparum infections [9, 10]. Earlier it was assumed a lower affinity of monoclonal antibodies for *P.* *ovale* *spp* and *P.* *malariae* antigens as an additional cause of reduced RDT sensitivity [11]. Current RDTs are not capable of differentiating infections with non-falciparum species as they are based on lactate dehydrogenase (LDH) and aldolase, common to all *Plasmodium* species [9, 12]. It was shown previously that *P.* *knowlesi* LDH can cross-react with that of *P.* *falciparum* and *P.* *vivax* [13]. However, molecular methods are very helpful to overcome the drawbacks of LM and RDTs. For instance, the limitations of LM compared to current molecular methods have been demonstrated to differentiate *P.* *falciparum*, *P.* *vivax*, and *P.* *knowlesi* in Sabah, Malaysia [8]. Molecular methods also allow the division of *P.* *ovale* *spp.* into two genetically distinct and morphologically similar subspecies: *P.* *ovale* *curtisi* (classic type) and *P.* *ovale* *wallikeri* (variant) [14]. Besides, molecular methods are very useful to track submicroscopic infections that represent a big challenge to successful malaria control [15, 16]. Thus, the real distribution of malaria species is not clearly established in endemic areas especially in sSA region. Furthermore, non-falciparum species are of great concern as recent reports have shown on their ability to elicit severe malaria attacks [17, 18].

As most of studies were conducted in Centre, Littoral, and Southwest regions there is paucity of studies on the molecular method-based distribution of *Plasmodium* species in Cameroon (Table 1). There is a growing number of reports on non-falciparum species circulation in Cameroon indicating the importance of non-falciparum species to define baseline epidemiological data and sensitize the general public/policy makers for effective malaria control programmes. Hence, this study aimed to characterize *Plasmodium* species especially non-falciparum species and determine the prevalence of submicroscopic malaria infections, in different facets of Cameroon.

**Table 1:** Landscape of molecular epidemiology studies on non-falciparum species in Cameroon

Study sites (Regions)	Period	Study population	Prevalence of *Plasmodium* species either mono-infection or co-infection	References
Bangolan (NW)	Sept–Nov 2007	Children/Pregnant women	*P.* *falciparum/P.* *malariae* (72.86%), *P.* *falciparum/P.* *malariae/P.* *ovale* (11.43%), *P.* *malariae* (5.71%), *P.* *vivax* (0%), *P.* *knowlesi* (NI)	[19]
Yaoundé (CEN) and eight villages around Yaoundé	–	Children and adults	*P.* *falciparum* (98.56%), *P.* *malariae* (6.26%)*,* *P.* *ovale* (0.02%) and *P.* *vivax* (0%)^¶^ *P.* *falciparum* (79.9%), *P.* *falciparum/P.* *malariae* (14.0%), *P.* *falciparum/P.* *ovale* (3.0%), *P.* *falciparum/P.* *malariae/P.* *ovale* (2.1%), *P.* *ovale* (0.9%)*,* *P.* *malariae* (0.2%) and *P.* *vivax* (0%)^¶¶^	[20]^§^
Bolifamba (SW)	Jul 2008–Oct 2009	Apparently healthy adults	*P.* *falciparum* (27.5%), *P.* *vivax* (3.3%), *P.* *malariae* (1.5%), *P.* *ovale* (0%), *P.* *falciparum/P.* *vivax* (1.1%), *P.* *falciparum/P.* *malariae* (1.5%), *P.* *falciparum/P.* *vivax/P.* *malariae* (0.4%), *P.* *knowlesi* (NI)	[21]
Douala (LIT), Ebolowa (S), Kye-Ossi (S), Yaoundé (CEN), Bertoua (E)	NA	Children and adults	*P.* *falciparum* (96%), *P.* *vivax* (3%), *P.* *falciparum/P.* *vivax* (1%), *P.* *knowlesi* (NI)	[22]
Nkassomo (CEN), Vian (CEN)	Feb–Mar 2011	Children and adults	*P.* *falciparum* (100%), *P.* *vivax* (0%), *P.* *malariae* (0%), *P.* *ovale* (0%), *P.* *knowlesi* (NI)	[23]
Douala (LIT)	NA	Children and adults	*P.* *falciparum* (76.7%), *P.* *vivax* (23.3%), *P.* *malariae* (0%), *P.* *ovale* (0%), *P.* *knowlesi* (NI)	[24]
Maroua (FN), Ngaoundere (ADA), Yaoundé (CEN), Bamenda (NW), Limbe (SW)	May–Nov 2015	Febrile children	*P.* *falciparum* (100%), *P.* *vivax* (0%), *P.* *malariae* (0%), *P.* *ovale* (0%), *P.* *knowlesi* (0%)	[25]
Dschang (W)	NA	Children and adults	*P.* *falciparum* (60%), *P.* *vivax* (35.8%), *P.* *ovale* (0%), *P.* *malariae* (1.4%), *P.* *falciparum/P.* *vivax* (2.8%), *P.* *knowlesi* (NI)	[26]
Mvan (CEN), Yaoundé (CEN)	NA	Asymptomatic children (3–14 yrs)	*P.* *falciparum* (87.9%), *P.* *vivax* (0%), *P.* *malariae* (4.8%), *P.* *ovale* (0%), *P.* *falciparum/P.* *malariae* (7.2%), *P.* *knowlesi* (NI)	[27]
Mutengene (SW)	Apr–Jun 2013	Children (6 mos–10 yrs)	*P.* *falciparum* (82.17%), *P.* *vivax* (0%), *P.* *ovale* (5.14%), *P.* *falciparum/P.* *ovale* (4.67%), *P.* *falciparum/P.* *malariae* (7.41%), *P.* *knowlesi* (NI)	[28]
Pitoa (N), Mayo-Oulo (N)	Nov 2014	Children (6 mos–10 yrs)	*P.* *falciparum* (86.6%), *P.* *vivax* (0%), *P.* *ovale* (0%), *P.* *malariae* (8.4%), *P.* *falciparum/P.* *malariae* (5%), *P.* *knowlesi* (NI)	[29]
Dschang (W)	NA	Children and adults^&^	*P.* *vivax* (35.4%)	[30]
Douala (LIT)	Aug–Sep 2018	Children and adults	*P.* *ovale* *curtisi* (five samples were PCR *P.* *ovale* *curtisi* positive/RDT negative)	[31]^†^
Tibati (ADA)	June–July 2015	Children and adults	In health centers [*P.* *falciparum* (98.8%), *P.* *malariae* (0.6%), *P.* *ovale* (0.6%), *P.* *vivax* (0%), *P.* *knowlesi* (NI), Co-infections (0%)]; In community [*P.* *falciparum* (76.4%), *P.* *malariae* (6.8%), *P.* *ovale* (0.2%), *P.* *vivax* (0%), *P.* *knowlesi* (NI), Co-infections (16.6%)]	[32]^‡^
Tibati (ADA) and Mfou (CEN)	July–August 2019 (Tibati) June–July 2018 (Mfou)	Children and adults	Tibati: *P.* *falciparum* (98.0%), *P.* *malariae* (0%), *P.* *vivax* (0%), *P.* *ovale* (1.5%), *P.* *falciparum/P.* *ovale* (0.5%), *P.* *knowlesi* (NI) Mfou: *P.* *falciparum* (95.8%), *P.* *malariae* (1.9%), *P.* *vivax* (0%), *P.* *ovale* (0%), *P.* *falciparum/P.* *malariae* (2.3%), *P.* *knowlesi* (NI)	[33]
Nkolbisson (CEN)	November 2019–February 2020	Children	*P.* *falciparum* (66.9%), *P.* *malariae* (5.5%) and *P.* *ovale* (3.1%)#	[34]
Five villages of the Esse District (CEN)	November–December 2018	Children and adults	*P.* *falciparum* (85.4%), *P.* *malariae* (2.1%), *P.* *ovale* (1.5%), *P.* *vivax* (0%), *P.* *falciparum/P.* *malariae* (8.5%), *P.* *falciparum/P.* *ovale* (1.8%), *P.* *falciparum/P.* *malariae/P.* *ovale* (0.6%), *P.* *knowlesi* (NI)	[35]
Douala (LT), Buea (SW)	2003–2005 and 2009–2013	Children and adults	In Douala: *P.* *falciparum* (84.9%), *P.* *falciparum/P.* *malariae* (8.1%), *P.* *falciparum/P.* *ovale* (7%) In Buea: *P.* *falciparum* (84.4%), *P.* *malariae* (5.2%), *P.* *falciparum/P.* *malariae* (10.4%), *P.* *knowlesi* (NI)	[36]

*ADA* Adamawa, *CEN* Centre, *E* East, *FN* Far North, *LIT* Littoral, *N* North, *NW* Northwest, *S* South, *SW* Southwest, *W* West, *NA* Not available, *MOS* Months, *NI* Not investigated, *RDT* Rapid diagnostic test, *Yrs* Years

Direct sequencing and Deep sequencing (barcoded pyrosequencing) of a 405-bp mitochondrial region of *Plasmodium* genome were used to analyze 77 samples (¶) and 437 samples (¶¶), respectively

^§^The samples were collected from eight villages close to the habitat of wild living *Plasmodium* infecting apes and the town of Yaoundé in HIV-infected patients

^&^The patients were all febrile and Duffy-negative

^†^This subspecies was found in five *P.* *falciparum* detecting-only RDT-based false negative results

^‡^The details on co-infections were not specified

^#^The prevalence of each species was determined using the total number of enrolled children (*n* = 127)

## Materials and methods

### Study design and population

**Fig. 1:**
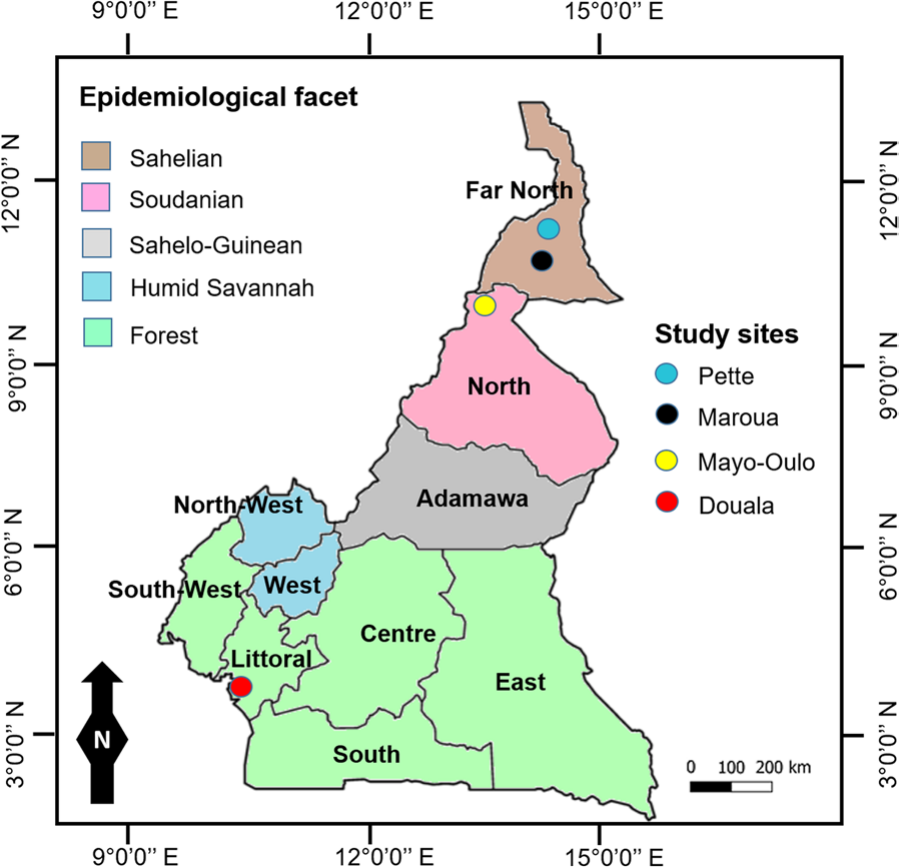
Figure showing epidemiological facets and study site locations in Cameroon

A multicentre cross-sectional study took place from August to October 2019 at health facilities in four towns located in three different malaria epidemiological strata/facets of Cameroon (Central Africa) viz.: (1) Sahelian (Pette, and Maroua), (2) Soudanian (Mayo-Oulo), and (3) Forest (Douala) (Fig. 1).

Maroua and Pette are characterized by dry and humid climate, and rainfall < 700 mm/year. Malaria is hyperendemic and seasonal transmission with epidemic outbreaks during rainy season for 2–3 months [37, 38]. Mayo-Oulo is characterized by annual rainfall 1000 mm/year, rainy season of 3–5 months, and a set of vegetation types (steppes, savannahs, shrubs, gallery forests). In Soudanian facet, malaria is hyperendemic and seasonal with epidemic patterns [37, 39]. Douala, the economic capital of Cameroon, is located in forest facet where malaria transmission is holoendemic and perennial. This facet is also characterized by diverse ecosystems (humid savannah, dense vegetation and large forests), hot and humid climate and heavy rainfall (between 1500 and 5000 mm^3^ each year) [37, 39]. The entomological inoculation rates vary from ~ 10 infective bites/man/month in Soudanian and Sahelian facets to 100 infective bites/man/month in forest facet [39].

All patients attending any of the four selected health facilities were included in the study. Inclusion criteria were defined as being resident in the study area with no travel history in the last three weeks and with or without fever. All patients who met the inclusion criteria and voluntarily agreed to participate in the study were recruited.

Before enrolment and administration of the questionnaire, adolescents, adult patients and parents/guardians of minors were informed about the purpose and process of investigation (background, goals, methodology, study constraints, data confidentiality, and rights to opt-out from the study), and signed informed consent was obtained from patients and children’s parents/guardians in accordance with Helsinki Declaration.

The Lorentz’ formula was used to estimate the required minimum sample size: *n* = Z^2^ × p × (1-p)/d^2^, where *n* = sample size required, p = assumed prevalence of non-falciparum species, Z = statistic for the desired confidence level (1.96 for 95% confidence level) and d = accepted margin of error (0.05). Based on Table 1, the maximum value of non-falciparum species prevalence was 90% so, the minimum sample size was estimated as *n* = 128 [19]. However, due of logistics, 119 individuals were enrolled. This is well above 90% of the expected computed sample size.

A simple investigation form was used for data collection on gender, age, axillary temperature, LM-based parasitaemia, clinical symptoms, and malaria medication history. Blood drops were spotted onto Whatman filter (GE Healthcare Ltd., Amersham, UK) and air-dried for 15 min. *Plasmodium* DNA was extracted to perform molecular analyses.

This study was conducted following ethics directive related to research on humans in Cameroon, and administrative authorizations were obtained from each health facility. The study was also approved by Institutional ethics committee of ICMR-National Institute of Malaria Research (NIMR) (N°PHB/NIMR/EC/2020/55). Participation was voluntary, anonymous without compensation and all methods were carried out in accordance with relevant guidelines and regulations.

### Blood sampling and microscopic detection of malaria parasites

Three drops of blood were collected from finger prick of each patient and used for (i) thick and thin blood smears for LM-based malaria diagnosis and (ii) dried blood spot (DBS) on Whatman filter paper, air-dried for 15 min, and then stored at 4 °C until further molecular studies.

Thick and thin blood smears were performed as per standard protocols to estimate parasite density and malaria parasite speciation on samples by two experienced technicians regularly attending seminars on skills assessment, organized by Ministry of Public Health (Yaoundé, Cameroon) [40]. Thin smears were fixed using absolute methanol, stained with 10% Giemsa, and then air-dried for 30 min. Blood drop used to make thick smear was stirred in circular motion with the corner of another glass slide and left to dry for 15 min without fixative. Thereafter, thick smear was then stained with 10% Giemsa, washed with buffered water and air-dried for 30 min. *Plasmodium* parasite density was expressed as number of parasites per microliter of blood and was determined based on the number of counted parasites per 200 leukocytes, assuming a total leukocyte count of 8,000 cells/µL of whole blood [40].

### Species identification

#### Extraction of plasmodial DNA

Genomic DNA from DBS was extracted using a commercial kit (QIAGEN blood DNA extraction kit, Valencia, California, USA) following the manufacturer’s instructions. DNA was then eluted into 100 µL of elution buffer (10 mM Tris–HCl; 0.5 mM EDTA; pH 9.0), and stored at − 20 °C until use.

#### Molecular diagnosis of malaria species

**Fig. 2:**
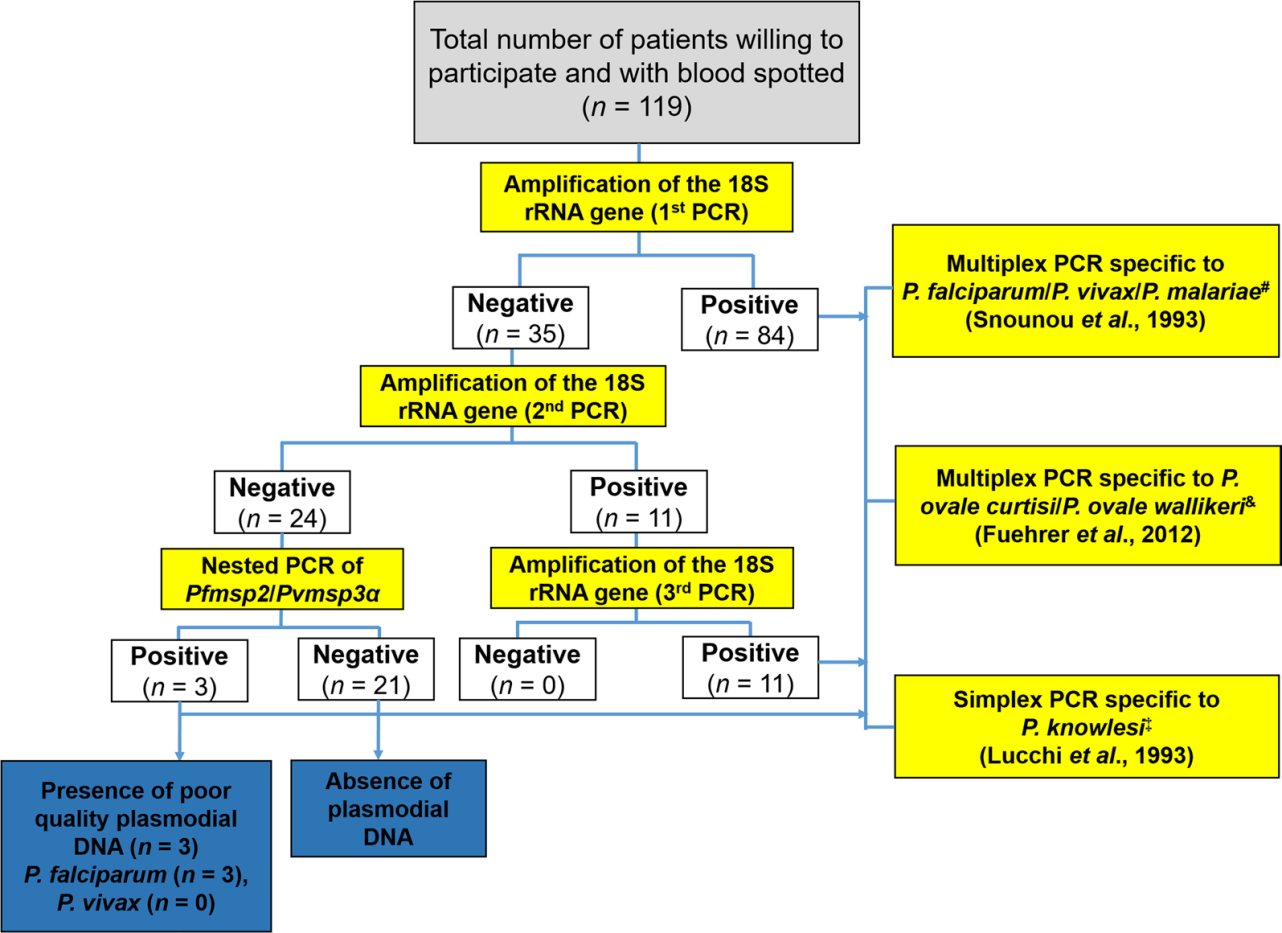
Flowchart depicting the process of molecular analyses. *DNA* deoxyribonucleic acid, *rRNA* ribosomal ribonucleic acid, *PCR* polymerase chain reaction; *msp* merozoite surface protein, *PCR* polymerase chain reaction. ^*^Primary PCR using specific primers for *Plasmodium* *spp.* species. ^#^Nested PCR using specific primers for *P.* *falciparum*, *P.* *vivax* and *P.* *malariae,* respectively ^&^Nested PCR using specific primers for *P.* *ovale* *curtisi* and *P.* *ovale* *wallikeri* respectively, ^‡^Nested PCR using specific primers for *P.* *knowlesi*.

The differentiation of human malarial species (*P.* *falciparum,* *P.* *vivax,* *P.* *ovale,* *P.* *malariae* and *P.* *knowlesi*) was performed using three PCR protocols: (i) a nested multiplex PCR for detection of *P.* *falciparum,* *P.* *vivax* and *P.* *malariae* [41, 42], (ii) a multiplex single step PCR for detection of *P.* *ovale* subspecies (*P.* *ovale* *curtisi* and *P.* *ovale* *wallikeri*) [43] and (iii) a simplex single step PCR for detection of *P.* *knowlesi* [44] (Fig. 2). Positive samples were considered positive after first PCR assay. Negative samples were repeated a second time to confirm again and PCRs were repeated a third time if results were discordant. The third PCR was performed using freshly extracted DNA from DBS to rule out any possibilities of contamination in samples.

Primers rPLU5 and rPLU6 were used to identify *Plasmodium* parasites at genus level, and then PCR products were used as template for identifying *Plasmodium* species using species-specific primers (Additional file 1). For example, PCR of *P.* *knowlesi* was made in a final volume of 25 µL using primers Pkr140-5F and Pkr140-5R. The PCR cycling conditions were initial denaturation (95 °C/2 min), followed by 35 cycles [denaturation: 95 °C/30 s, annealing: 57 °C/30 s, and extension: 72 °C/45 s], and a final extension (72 °C/5 min). *P.* *knowlesi* infection was confirmed by presence of 200 bp PCR product on electrophoresis gel [44]. DNA sample was mixed in 25 µL PCR reaction containing 2.5 µL of Taq Buffer A (GeNei™, India), 2 µL of dNTPs mix (10 mM, GeNei™, India), 1 µL of each primer (10 µM), 0.3 unit of Taq polymerase (GeNei™, India), 2 µL DNA template and free-nuclease water Q.S. Controls were run at each PCR.

Amplicons were loaded on 2% agarose gel pre-stained with ethidium bromide for gel electrophoresis. The electrophoretic migration of amplicons was performed at 72 V for 1 h. The amplicons were visualized using an UV trans-illuminator. To ascertain that negative *18S* *SSU* *rRNA* gene PCR results were not due to poor quality DNA, the single copy merozoite surface protein 2 (*msp2*) gene was amplified (Additional file 1) [45]. In case of negative result, sample was considered as lacking plasmodial DNA. Conversely, samples were considered as having bad quality plasmodial DNA in case of positive result (Fig. 2).

#### Further characterization of non-falciparum species

The exon 4 of glucose-6-phosphate dehydrogenase gene (G6PD, NC_000023.11) was amplified and genetic polymorphism of G6PD gene was evaluated for 202A mutation by sequencing (Additional file 1) [46]. This mutation is one of the most frequently and more commonly associated with G6PD deficiency (G6PD-d), and is a serious impediment to use of primaquine (PQ) for radical cure of non-falciparum malaria in sSA populations [47].

#### Sequencing and phylogeny

PCR products of non-falciparum samples were purified using DNA GeneJET PCR purification kit (ThermoFisher Scientific, Lithuania), and then sequenced on an ABI3037XL genetic analyser (Applied Biosystems) with 2X coverage (sequenced from both forward and reverse directions). BLAST analysis of the *18S* *RNA* and *G6PD* genes was performed using MEGA X software version 10.0.5 [48]. We compared 18S sequences of non-falciparum species identified with those of previous isolates from different geographical areas through NCBI website (www.ncbi.nlm.nih.gov). Nucleotide and deduced amino acid sequences were aligned using CLUSTALW algorithm [48].

The evolutionary history was inferred using Neighbour-Joining method [49]. A bootstrap consensus tree inferred from 1000 replicates was taken to represent evolutionary history of the taxa analysed. Branches corresponding to partitions reproduced in less than 50% bootstrap replicates were collapsed. Percentage of replicate trees in which the associated taxa clustered together was computed using bootstrap test (1000 replicates). Evolutionary distances were computed using maximum composite likelihood method. The rate variation among sites was modelled with a gamma distribution.

### Operational definitions

Fever was defined as axillary temperature ≥ 37.5 °C [50]. Parasitemia not detected using LM but detected by PCR were defined as submicroscopic infections while those detected by LM and PCR were defined as microscopic infections [51]. Discordance between LM and PCR results were finally decided positive or negative based on PCR result. Asymptomatic infection was defined as presence of malaria parasites in absence of fever while symptomatic infection was defined as presence of parasites with fever [50].

### Statistical analysis

Data were keyed in an Excel spreadsheet (Microsoft Office 2016, USA), coded, and checked for consistency. Qualitative variables were presented as frequency, percentage, and confidence interval at 95% (95% CI) while quantitative variables were presented as mean ± standard deviation (SD) [52]. Independence Pearson’s chi-square and Fisher’s exact tests were used to compare proportions. Unpaired t, Mann–Whitney, and Kruskal–Wallis tests were used to compare mean values of parasitaemia with respect to categorical variables. Pearson correlation test was also performed to test association between parasitaemia and age. Parasitaemia were log_10_-transformed before statistical analysis. The level of statistical significance was set at *p-value* < 0.05. Statistical analysis was performed using the statistical package for social sciences v16 for Windows (SPSS, IBM, Chicago, Illinois, USA) and StatView v5.0 for Windows (SAS Institute, Inc., Chicago, Illinois, USA).

## Results

### Baseline characteristics of patients

The study population was mainly composed of females (60.7%) (Table 2). The mean age of patients was 19.09 ± 14.64 years (range: 2 months–64 years). Children aged < 5 years accounted for 18.8% of all patients. No statistically significant difference was found between gender and age groups, even though proportions of males were higher than those of females in age groups of < 5 years (23.9% *vs* 15.4%), 5–10 years (13.0% *vs* 12.7%), 10–15 years (17.4% *vs* 14.1%), 30–35 years (8.7% *vs* 8.5%), and ≥ 35 years (17.4% *vs* 16.8%) (χ^2^ = 4.015, df = 7, *p* = 0.77). The same pattern was observed between gender and age with respect to malaria epidemiological facet.

**Table 2:** Baseline characteristics of participants

Facet	Age (years)								Total
	< 5	[5–10]	[10–15]	[15–20]	[20–25]	[25–30]	[30–35]	≥ 35	
*Sahelian*									
Female	5 (14.3)	4 (11.4)	6 (17.2)	4 (11.4)	5 (14.3)	5 (14.3)	2 (5.7)	4 (11.4)	35 (60.3)
Male	3 (13.0)	4 (17.4)	5 (21.8)	2 (8.7)	1 (4.3)	1 (4.3)	2 (8.7)	5 (21.8)	23 (39.7)
Total	8 (13.8)	8 (13.8)	11 (19.1)	6 (10.3)	6 (10.3)	6 (10.3)	4 (6.9)	9 (15.5)	58
*Forest*									
Female	4 (14.8)	3 (11.1)	3 (11.1)	3 (11.1)	3 (11.1)	0 (0.0)	4 (14.8)	7 (26.0)	27 (58.7)
Male	5 (26.3)	2 (10.5)	3 (15.8)	2 (10.5)	1 (5.3)	1 (5.3)	2 (10.5)	3 (15.8)	19 (41.3)
Total	9 (19.6)	5 (10.9)	6 (13.0)	5 (10.9)	4 (8.7)	1 (2.2)	6 (13.0)	10 (21.7)	46
*Soudanian*									
Female	2 (22.2)	2 (22.2)	1 (11.1)	0 (0.0)	2 (22.2)	1 (11.1)	0 (0.0)	1 (11.1)	9 (69.2)
Male	3 (75.0)	0 (0.0)	0 (0.0)	0 (0.0)	0 (0.0)	1 (25.0)	0 (0.0)	0 (0.0)	4 (30.8)
Total	5 (38.4)	2 (15.4)	1 (7.7)	0 (0.0)	2 (15.4)	2 (15.4)	0 (0.0)	1 (7.7)	13
*All*									
Female	11 (15.4)	9 (12.7)	10 (14.1)	7 (9.9)	10 (14.1)	6 (8.5)	6 (8.5)	12 (16.8)	71 (60.7)
Male	11 (23.9)	6 (13.0)	8 (17.4)	4 (8.7)	2 (4.3)	3 (6.5)	4 (8.7)	8 (17.4)	46 (39.3)
Total	22 (18.8)	15 (12.8)	18 (15.4)	11 (9.4)	12 (10.3)	9 (7.7)	10 (8.5)	20 (17.1)	117*

Data are presented as frequency (percentage), *Data were missing for two patients

### Malaria prevalence

Based on PCR results, the overall malaria prevalence was 82.4% (98/119; 95% CI 74.5–88.2%). Out of 86 LM-positive samples, PCR detected malaria parasites in 84 of them. Among 33 LM-negative samples, PCR-detected malaria parasites in 14 samples (Additional file 2). Thus, sensitivity and specificity of LM were 85.7% (95% CI 77.4–91.2%) and 90.5% (95% CI 71.1–97.3%), respectively. Diagnosis with both techniques were largely in agreement with a kappa index of 0.70 based on classification defined by Landis and Koch [53]. It should be noted that all non-falciparum infections detected by nested PCR were misdiagnosed as *P.* *falciparum* by thin smear.

### Prevalence of malaria infection by gender and age

The relationship between malaria prevalence, sociodemographic characteristics and epidemiological facet is shown in Additional file 3. No statistically significant association was observed between malaria infection and age in all epidemiological facets. Likewise, no association was found between malaria infection and epidemiological facet (χ^2^ = 0.552, df = 1, *p* = 0.75), although the prevalence of malaria was highest in Soudanian facet (84.6%). However, malaria infection varied significantly according to gender, with higher malaria prevalence reported in males when compared to females from Forest facet (94.7% *vs* 71.4%, *p* = 0.04) (Additional file 3). To be noted, high proportion of asymptomatic malaria carriage (10.8%) was found in the present study.

There was no statistically significant difference between parasitemia with respect to gender (t-test: *p-*value = 0.30) and age (Kruskal–Wallis test: H = 6.819; *p* = 0.11). A non-significant negative correlation was found between parasitaemia and age (r = − 0.204; 95%CI − 0.149 to 0.032, *p* = 0.08). Parasitaemia was significantly higher in Forest facet (Douala) when compared to samples from Sahelian facet (Pette) (3.745 ± 0.884 versus 2.984 ± 0.336 parasites/µL of blood, Mann–Whitney test: *p* = 0.0001).

### *Plasmodium* species identification

**Fig. 3:**
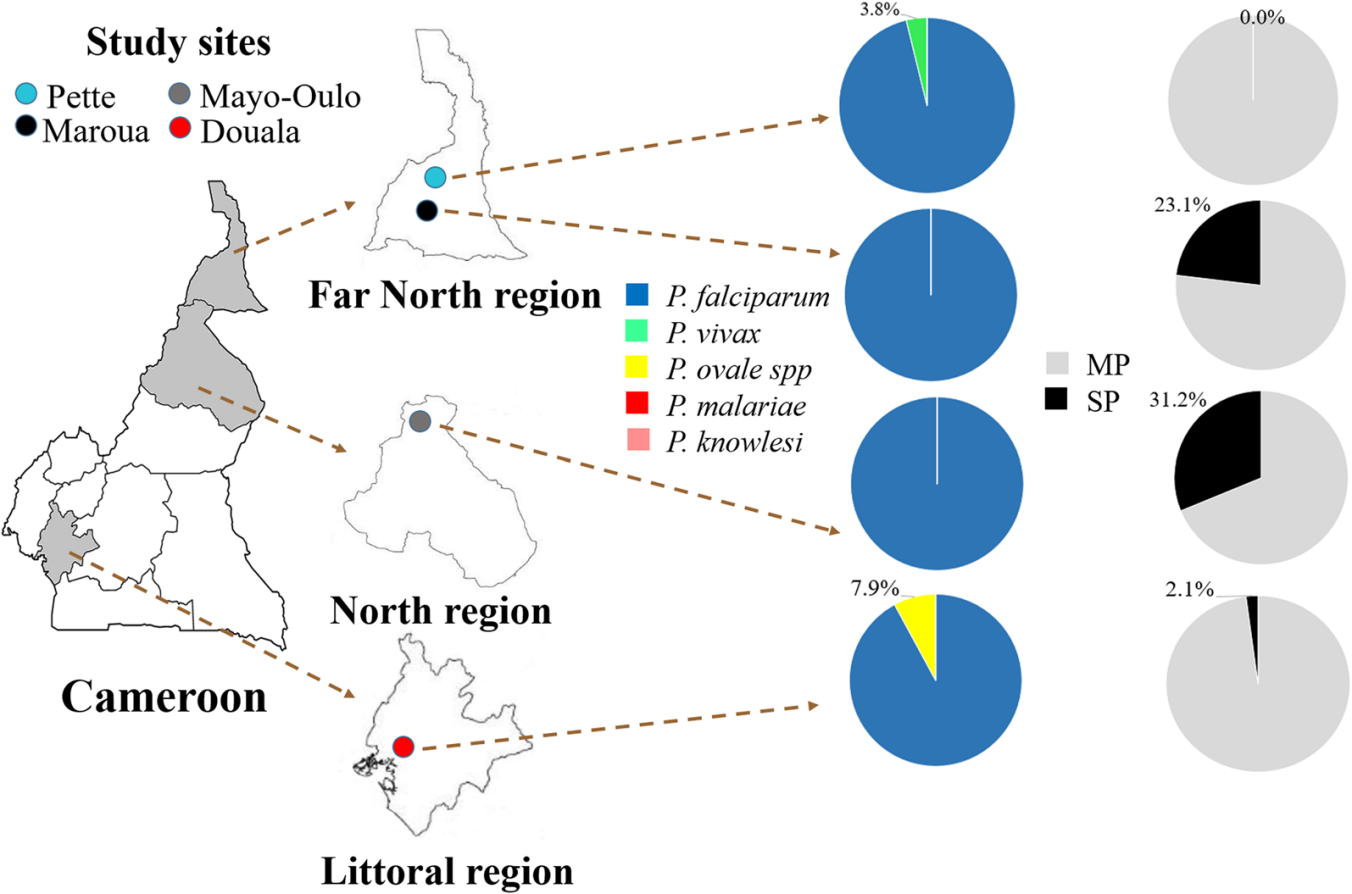
Proportion of *Plasmodium* species and submicroscopic infections in different study sites. MP: Microscopic parasitemia, SP: Submicroscopic parasitemia

Using nested PCR, three *Plasmodium* species were found among 98 infection cases viz. *P.* *falciparum*, *P.* *ovale* *curtisi*, and *P.* *vivax*. Two types of co-infections, *P.* *falciparum* + *P.* *vivax*, and *P.* *falciparum* + *P.* *ovale* *curtisi* were recorded. The remaining infections were mono-infections as either *P.* *falciparum* or *P.* *ovale* *curtisi*. The prevalence of each species was as follows: *P.* *falciparum* (*n* = 96; 96.9%), *P.* *ovale* *curtisi* (*n* = 3; 3.0%), and *P.* *vivax* (*n* = 1; 1.0%). *P.* *falciparum* was the only species recorded in Maroua and Mayo-Oulo, while *P.* *ovale* *curtisi* and *P.* *vivax* were only found in Douala and Pette, respectively (Fig. 3). No cases of *P.* *malariae*, *P.* *ovale* *wallikeri* and *P.* *knowlesi* were found in this study.

### Submicroscopic malaria infections

**Fig. 4:**
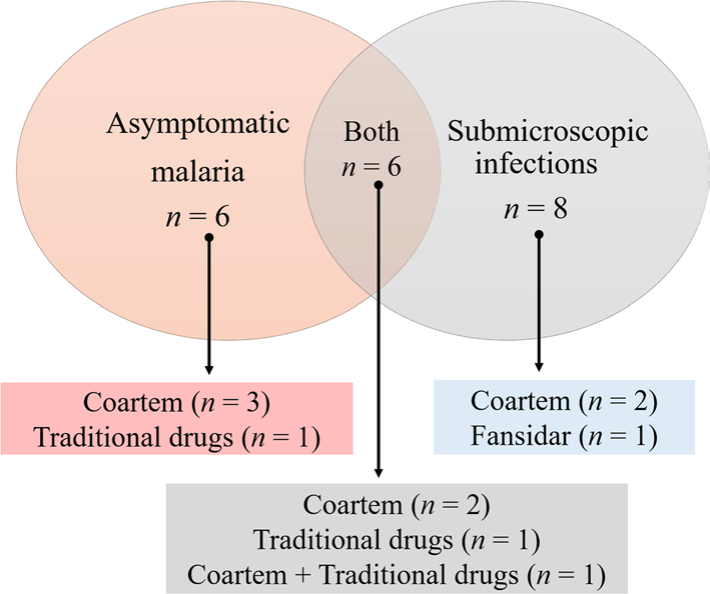
Venn diagram of asymptomatic and submiscroscopic infections with link to antimalarial self-medication

In this study, the overall proportion of submicroscopic infections was 11.8% (14/119, 95% CI 7.1–18.8%). These infections accounted for 14.3% (14/98) of all malaria infections. The prevalence of submicroscopic infections varied between areas, with the highest values found in Mayo-Oulo (31.2%, χ^2^ = 25.37, df = 3, *p* < 0.0001) (Fig. 3). We found six and eight individuals with asymptomatic and submicroscopic infections, respectively (Fig. 4). All non-falciparum infections were symptomatic with microscopic parasitaemia. Recent self-medication (i.e., drug taken < 7 days before consultation) with artemether + lumefantrine (Coartem®), sulfadoxine + pyrimethamine (Fansidar®), and plant-based traditional medicines was seen among few asymptomatic and/or submicroscopic infection cases (Fig. 4).

### Demography of asymptomatic and submicroscopic infections

The proportion of asymptomatic infections was higher among females (13.9%, 95% CI 6.72–22.08%) when compared to males (4.3%, 95% CI: 1.20–14.54%) but no statistically significant difference was found (χ^2^ = 2.207, df = 1, *p* = 0.19). Likewise, submicroscopic infections were more prevalent in females compared to males (15.3% vs 6.5%, *p* = 0.04). The proportion of asymptomatic and submicroscopic infections did not vary significantly according to patients’ age although proportions of these two types of infections were higher among those aged > 20 years (15.7%) and 5–10 years (13.3%), respectively.

### Characterization of *non-falciparum* species

All non-falciparum infections were symptomatic and microscopic with no G6PD–d case among non-falciparum infections (Table 3).

**Table 3:** Characteristics of *P.* *vivax* and *P.* *ovale* infections

Malaria species	Parasitemia (parasites/µL)	Malaria and parasitemia	G6PD status	Hospital treatment prescribed	Patient outcome
*P.* *ovale* *curtisi*^*a§*^	4870	Symptomatic and microscopic	G6PD-n	AS + AQ	Discharged
*P.* *ovale* *curtisi*^b**^	850	Symptomatic and microscopic	G6PD-n	AS + AQ	Discharged
*P.* *ovale* *curtisi*^c**^	1453	Symptomatic and microscopic	G6PD-n	AL	Discharged
*P.* *vivax*^*§*^	1500	Symptomatic and microscopic	G6PD-n	NA	NA

*AS + AQ* Artesunate + Amodiaquine, *AL* Artemether + Lumefantrine, *G6PD* Glucose-6-Phosphate Dehydrogenase, *G6PD-n* Glucose-6-Phosphate Dehydrogenase—No deficiency, *NA* not available;

*P.* *ovale* *curtisi* (samples a, b and c identified in the study)

^**^Mono-infection

^§^Co-infection with *P.* *falciparum*

### BLAST of non-falciparum species

The phylogenetic analysis outlined a genetic relatedness between *P.* *ovale* *curtisi* sequences that formed a cluster with NCBI-retrieved *P.* *ovale* *curtisi* sequences from India (Additional file 4). *P.* *vivax* sequence was phylogenetically closer to SAL-I reference strain and sequences from Nigeria, India, and Cameroon (Additional file 4).

## Discussion

The present study aimed at addressing the epidemiology of non-falciparum species and submicroscopic infections in three malaria epidemiological facets of Cameroon.

*P.* *falciparum* was the main species responsible for malaria infection in this study. Two other species namely *P.* *vivax* and *P.* *ovale* *curtisi* were also reported in this study. The presence of *P.* *vivax* in Cameroon has been reported at high prevalence (up to 35.8%) by previous studies, suggesting growing evidence of its circulation in the country [21, 22, 24, 26]. Interestingly, the *P.* *vivax* sample was found in Pette located in Sahelian facet of Cameroon. To the best of our knowledge, this is the first evidence of PCR- and sequencing-confirmed circulation of *P.* *vivax* in this area of Cameroon. *Plasmodium* *vivax* control is tricky due to several reasons: (i) this species has dormant stages—called hypnozoites—responsible for malaria relapses many months to years after the first infection; hypnozoites are resistant to antimalarial drugs, and constitute an invisible reservoir of *P.* *vivax* parasites [15]; (ii) continuous in vitro culture of *P.* *vivax* is difficult despite a few encouraging reports [54] making elusive the understanding of its biology and hindering malaria vaccine development; (iii) ability to infect Duffy-negative individuals and (iv) its interaction with other *Plasmodium* species can modulate malaria severity [55]. Also, increasingly research reports outline the ability of *P.* *vivax* to elicit severe malaria attacks and deaths [55–57].

*P.* *ovale* *curtisi* was found at low prevalence among study population. This study is the first to confirm clearly the presence of *P.* *ovale* subspecies in Cameroon. Current published and available data on these two subspecies in Cameroon come from a recent study on local infection [31] and imported malaria [58–60]. Zhou and colleagues outlined that prevalence of these subspecies is underestimated in sSA [59]. There is a need to address *P.* *ovale* species in Cameroon due to their role in life-threatening malaria complications viz. severe thrombocytopenia [58], severe renal failure, and acute respiratory distress syndrome [61].

This study also confirms the absence of *P.* *knowlesi* in Cameroon as reported in a previous study conducted in five areas of the country (Maroua, Ngaoundere, Yaoundé, Bamenda, and Limbe) [25]. This is a zoonotic species of South Asian macaques [62], and has been reported as important cause of human malaria in Malaysia, and seems to be limited in SEA so far [7]. A possible circulation of *P.* *knowlesi* in humans in sSA should not be ruled out given both high rate of human migration between individuals from sSA and SEA, and presence of available animal reservoirs in sSA. This hypothesis of human migration to explain the presence of imported *Plasmodium* populations in Cameroon is supported by our phylogenetic analyses. The *P.* *vivax* sample from Pette town formed a cluster with *P.* *vivax* strain of Nigerian origin. This town is located in the Far North region of Cameroon which is geographically bordered by several countries including Nigeria.

Keeping in mind the necessity to address non-falciparum species, this brings back on table the problem of using PQ in association with ACT or CQ in African individuals [63]. PQ is used in Asian and Latin American countries to clear *P.* *vivax* and *P.* *ovale* hypnozoites. Since PQ can induce haemolytic anaemia in G6PD–d persons, testing for G6PD status especially in children, must be performed prior to PQ treatment [63]. Testing for G6PD status is not implemented in Cameroon as it is thought that these two species circulate at low rate. In this study, G6PD-d was not found among patients infected with non-falciparum species suggesting the possibility to treat them with PQ.

Finally, the management of non-falciparum species is worth addressing in Cameroon, and it is crucial to achieve WHO’s elimination objectives [64]. A fact to support this assertion is prevalence of *P.* *ovale* and *P.* *malariae* reported in an area of declining *P.* *falciparum* transmission in Tanzania [65] thereby suggesting the possibility of shift from *P.* *falciparum* to non-falciparum species, if they are not addressed.

Submicroscopic infections were also reported in this study. PCR is more sensitive than LM, with a limit of detection as low as 5 parasites/µL of blood while that of LM is 50–500 parasites/µL of blood [66]. The implementation of PCR in health facilities is challenging as it is costly and requires a high level of expertise [50]. Most of submicroscopic infections (31.2%) were found in Mayo-Oulo (Sahelian facet). This finding agrees a meta-analysis study showing a higher submicroscopic infection prevalence in low transmission settings compared to high transmission sites [67, 68]. Submicroscopic infections are a real challenge to malaria control as they fuel malaria transmission and can occasionally lead to acute malaria [67].

Interestingly, self-medication practice with antimalarial and/or traditional drugs was reported in asymptomatic and/or submicroscopic infection cases. Self-medication is a commonly reported problem in Cameroonian population [69] which expose individuals to drug-related adverse effects and increase the risk for emergence and spread of drug-resistant *P.* *falciparum* parasites, especially to ACTs used as first line treatment [70]. Mostly, the African population attend health facilities after the disease still persists or gets worse after many self-management attempts. Thus, asymptomatic malaria and submicroscopic infection cases found in this study are likely due to self-medication. In this regard, it is also important to address self-medication with antimalarial drugs as some recent reports highlight independent emergence of ACT-resistant *P.* *falciparum* parasites in Rwanda and Uganda (East-central Africa), and *P.* *falciparum* parasites with ACT-resistance associated mutations in the *PfKelch13* gene in other parts of Africa (e.g., The Democratic Republic of Congo) [70–73].

## Conclusions

Malaria infection was highly prevalent with a predominance of *P.* *falciparum* irrespective of epidemiological facet. In addition, *P.* *ovale* *curtisi* and *P.* *vivax* were the two non-falciparum species found, while *P.* *knowlesi*, *P.* *ovale* *curtisi* and *P.* *malariae* were not seen in this study. Malaria prevalence varied with regard to epidemiological facet with highest burden in Forest facet. All non-falciparum species cases found were symptomatic and microscopic. Asymptomatic and submicroscopic infections accounted for > 10% of all infections, and were likely due to self-medication with antimalarials and/or traditional medicines. Greater attention should be given to non-falciparum species, asymptomatic/submicroscopic infections, and self-medication in Cameroon to successfully achieve malaria control and elimination programmes.

## Supplementary Information

**Additional file 1.** Primers used to discriminate human *Plasmodium* species, and amplify the *msp2* and *G6PD* genes.

**Additional file 2.** Performance of Giemsa-based microscopy incomparison with nested PCR in the identification of malaria infection.  

**Additional file 3.** Malaria infection by age, gender and strata.  

**Additional file 4.** Neighbor-Joining tree of *P. ovale* subspecies and *P.vivax* in comparison to NCBI sequences based on 18S rRNA gene sequences andreference strains. 

## Data Availability

All the data supporting the study findings are within the manuscript. Additional detailed information and raw data will be shared upon request addressed to the corresponding author.

## Abbreviations

ACT: Artemisinin-based combination therapy
CI: Confidence interval
DNA: Deoxyribonucleic acid
EDTA: Ethylenediaminetetraacetic acid
EtBr: Ethidium bromide
DBT: Department of Biotechnology
df: Degree of freedom
dNTP: Deoxynucleotide triphosphate
DBP: Duffy binding protein
EBP: Erythrocyte binding protein
FN: False negative
FP: False positive
FPR: False positive rate
FNR: False negative rate
ICMR: Indian Council of Medical Research
LDH: Lactate dehydrogenase
MARA: Mapping malaria risk in Africa
MOS: Months
MSP: Merozoite surface protein
NA: Not available
NI: Not investigated
NIMR: National Institute of Malaria Research
NPV: Negative predictive value
PCR: Polymerase chain reaction
Pos: Positive
PPV: Positive predictive value
PQ: Primaquine
Q.S: *Quantum* *satis*
RDT: Rapid diagnostic test
Ref: Reference
RNA: Ribonucleic acid
SD: Standard deviation
Se: Sensitivity
Sp: Specificity
sSA: Sub-Saharan Africa
SSU: Small subunit
TN: True negative
TP: True positive
TWAS: The World Academy of Sciences
WHO: World Health Organization
