# Supplementary material for: Non-falciparum species and submicroscopic infections in three epidemiological malaria facets in Cameroon
Source: BMC Infect Dis. 2022 Dec 2;22:900. doi: 10.1186/s12879-022-07901-6 (PMC9718470; doi:10.1186/s12879-022-07901-6)
Supplement: Supplementary file 1 — Additional file 1. Primers used to discriminate human Plasmodium species, and amplify the msp2 and G6PD genes. [file 12879_2022_7901_MOESM1_ESM.docx]

**Additional file 1**. Primers used to discriminate human *Plasmodium* species, and amplify the *msp2* and *G6PD* genes

|  |  | **Denaturation** | |  | **Annealing** | |  | **Elongation** | |  |  |  |
| --- | --- | --- | --- | --- | --- | --- | --- | --- | --- | --- | --- | --- |
| **Genes** | **Primer sequences (5' - 3')** | Tem (°C) | Time (min/sec) |  | Tem (°C) | Time (min/sec) |  | Tem (°C) | Time (min/sec) |  | **Cycles** | **Amplicon**  **Size (bp)** |
|  | **Primary PCR** |  |  |  |  |  |  |  |  |  |  |  |
| *18S* | rPLU5: CCTGTTGTTGCCTTAAACTTC | 94 | 1 |  | 58 | 2 |  | 72 | 2 |  | 24 | 1200 |
|  | rPLU6: TTAAAATTGTTGCAGTTAAAACG |  |  |  |  |  |  |  |  |  |  |  |
|  | **Nested PCR^a^** |  |  |  |  |  |  |  |  |  |  |  |
| *Pf 18S* | rFAL1: TTAAACTGGTTTGGGAAAACCAAATATATT | 94 | 1 |  | 58 | 2 |  | 72 | 2 |  | 24 | 205 |
|  | rFAL2: ACACAATGAACTCAATCATGACTACCCGTC | |  |  |  |  |  |  |  |  |  |  |
| *Pv 18S* | rVIV1: CGCTTCTAGCTTAATCCACATAACTGATAC | 94 | 1 |  | 58 | 2 |  | 72 | 2 |  | 24 | 120 |
|  | rVIV2: ACTTCCAAGCCGAAGCAAAGAAAGTCCTTA |  |  |  |  |  |  |  |  |  |  |  |
| *Pm 18S* | rMAL1: ATAACATAGTTGTACGTTAAGAATAACCGC | 94 | 1 |  | 58 | 2 |  | 72 | 2 |  | 24 | 144 |
|  | rMAL2: AAAATTCCCATGCATAAAAAATTATACAAA | |  |  |  |  |  |  |  |  |  |  |
|  | **Nested PCR^b^** |  |  |  |  |  |  |  |  |  |  |  |
| *Pk 18S* | Pkr140-5F: CAGAGATCCGTTCTCATGATTTCCATGG | 95 | 30 s |  | 57 | 30s |  | 72 | 45s |  | 35 | 200 |
|  | Pkr140-5R: CTRAACACCTCATGTCGTGGTAG |  |  |  |  |  |  |  |  |  |  |  |
|  | **Nested PCR^c^** |  |  |  |  |  |  |  |  |  |  |  |
| *PoC 18S* | rOVA1: ATCTCTTTTGCTATTTTTAGTATTGGAGA | 94 | 1 |  | 58 | 2 |  | 72 | 2 |  | 24 | 800 |
|  | rOVA2: GGAAAAGGACACATTAATTCTGTATCCTAGTG |  |  |  |  |  |  |  |  |  |  |  |
| *PoW 18S* | rOVA1v: ATCTCCTTTACTTTTTGTACTGGAGA | 94 | 1 |  | 58 | 2 |  | 72 | 2 |  | 24 | 782 |
|  | rOVA2v: GGAAAAGGACACTATAATGTATCCTAATA |  |  |  |  |  |  |  |  |  |  |  |
|  | **Primary PCR** |  |  |  |  |  |  |  |  |  |  |  |
| *msp2* | M2-OF: ATGAAGGTAATTAAAACATTGTCTATTATA | 95 | 1 |  | 58 | 2 |  | 72 | 2 |  | 30 | Variable |
|  | M2-OR: CTTTGTTACCATCGGTACATTCTT |  |  |  |  |  |  |  |  |  |  |  |
|  | **Nested PCR** |  |  |  |  |  |  |  |  |  |  |  |
| IC/3D7 | M2-ICF: AGAAGTATGGCAGAAAGTAAKCCTYCTACT | 95 | 1 |  | 61 | 2 |  | 72 | 2 |  | 30 | Variable |
|  | M2-ICR: GATTGTAATTCGGGGGATTCAGTTTGTTCG |  |  |  |  |  |  |  |  |  |  |  |
| FC27 | M2-FCF: AATACTAAGAGTGTAGGTGCARATGCTCCA | 95 | 1 |  | 61 | 2 |  | 72 | 2 |  | 30 | Variable |
|  | M2-FCR: TTTTATTTGGTGCATTGCCAGAACTTGAAC |  |  |  |  |  |  |  |  |  |  |  |
|  | **Single step PCR** |  |  |  |  |  |  |  |  |  |  |  |
| G6PD | G6F: GTGGCTGTTCCGGGATGGCCTTCTG | 94 | 1 |  | 58 | 1 |  | 72 | 1 |  | 35 | 109 |
|  | G6R: CTTGAAGAAGGGCTCACTCTGTTTG |  |  |  |  |  |  |  |  |  |  |  |

G6PD: Glucose-6-Phosphate dehydrogenase, *msp2*: Merozoite surface protein 2

^a^The primers were used to differentiate between *P. falciparum*, *P. vivax* and *P. malariae*

^b^The primers were used to track *P. knowlesi*

^c^The primers rOVA1 and rOVA2 were used to differentiate *P. ovale curtisi* while primers rOVA1v and rOVA2v were used to detect *P. ovale wallikeri*
