# Supplementary material for: Non-falciparum species and submicroscopic infections in three epidemiological malaria facets in Cameroon
Source: BMC Infect Dis. 2022 Dec 2;22:900. doi: 10.1186/s12879-022-07901-6 (PMC9718470; doi:10.1186/s12879-022-07901-6)
Supplement: Supplementary file 2 — Additional file 2. Performance of Giemsa-based microscopy incomparison with nested PCR in the identification of malaria infection. [file 12879_2022_7901_MOESM2_ESM.docx]

**Additional file 2**. Performance of Giemsa-based microscopy in comparison with nested PCR in the identification of malaria infection

|  |  | **nPCR** | |  |
| --- | --- | --- | --- | --- |
|  |  | Negative | Positive | **Total** |
| **LM** | Negative | 19 (TN) | 14 (FN) | **33** |
|  | Positive | 2 (FP) | 84 (TP) | **86** |
|  | **Total** | **21** | **98** | **119** |
|  |  |  |  |  |
| Se, % (95% CI) | 85.7 (77.4 - 91.2) |  |  |  |
| Sp, % (95% CI) | 90.5 (71.1 - 97.3) |  |  |  |
| PPV, % (95% CI) | 97.6 (91.9 – 99.3) |  |  |  |
| NPV, % (95% CI) | 57.6 (40.8 – 72.8) |  |  |  |
| FNR, % (95% CI) | 14.3 (8.7 – 22.6) |  |  |  |
| FPR, % (95% CI) | 9.5 (2.7 – 28.9) |  |  |  |
| Accuracy, %, (95% CI) | 86.6 (79.3 – 91.6) |  |  |  |
| Kappa (Standard error), *p-value* | 0.70 (0.07), < 0.0001* |  |  |  |

LM: Light microscopy, PCR: Nested polymerase chain reaction, FN: False negative, FP: False positive, TN: True negative, TP: True positive, Se: sensitivity, Sp: Specificity, PPV: Positive predictive value, NPV: Negative predictive value, FPR: False positive rate, FNR: False negative rate, 95%CI: confidence interval at 95%, NS: Not statistically significant, Se = [(TP/TP + FN)*100], Sp = [(TN/TN + FP)*100], PPV = [(TP/TP + FP)*100], NPV = [(TN/TN + FN)*100], FPR = 100 – Sp, FNR = 100 – Se, Kappa index were computed and categorized as described by Landis and Koch as follows: very poor (<0.00), poor (0.00-0.20), moderate (0.21-0.60), good (0.61-0.80) and excellent (≥ 0.81); *: statistically significant at *p* < 0.05.
