# Supplementary material for: Non-falciparum species and submicroscopic infections in three epidemiological malaria facets in Cameroon
Source: BMC Infect Dis. 2022 Dec 2;22:900. doi: 10.1186/s12879-022-07901-6 (PMC9718470; doi:10.1186/s12879-022-07901-6)
Supplement: Supplementary file 3 — Additional file 3. Malaria infection by age, gender and strata. [file 12879_2022_7901_MOESM3_ESM.docx]

**Additional file 3**. Malaria infection by age, gender and strata

|  | **Epidemiological facet** | | | | | | | |  |  |
| --- | --- | --- | --- | --- | --- | --- | --- | --- | --- | --- |
|  | **Forest** | |  | **Sahelian** | |  | **Soudanian** | |  |  |
| **Variables** | *n* | Pos (%) |  | *n* | Pos (%) |  | *n* | Pos (%) | ***χ^2^*** | ***p-value*** |
| **Gender** |  |  |  |  |  |  |  |  |  |  |
| Female | 28 | 20 (71.4%) |  | 35 | 32 (91.4%) |  | 9 | 7 (77.8%) | *5.239* | *0.07* |
| Male | 19 | 18 (94.7%) |  | 23 | 17 (73.9%) |  | 4 | 4 (100.0%) | *5.932* | *0.05* |
| ***χ^2^*** | *3.972* | |  | *3.248* | |  | *0.325* | |  |  |
| ***p-value*** | *0.046** | |  | *0.07* | |  | *0.56* | |  |  |
| **Age** |  |  |  |  |  |  |  |  |  |  |
| < 5 yrs | 8 | 6 (75.0%) |  | 8 | 7 (87.5%) |  | 5 | 5 (100.0%) | *0.409* | *0.81* |
| 5-10 yrs | 5 | 4 (80.0%) |  | 8 | 8 (100.0%) |  | 2 | 2 (100.0%) | *2.143* | *0.34* |
| 10-20 yrs | 11 | 9 (81.8%) |  | 17 | 13 (76.5%) |  | 1 | 1 (100.0%) | *0.387* | *0.82* |
| ≥ 20 yrs | 21 | 17 (84.0%) |  | 25 | 21 (84.0%) |  | 5 | 3 (60.0%) | *-* | *-* |
| ***χ^2^*** | *0.160* | |  | *2.362* | |  | *6.240* | |  |  |
| ***p-value*** | *0.98* | |  | *0.50* | |  | *0.10* | |  |  |

Data are presented as frequency and percentage; Pos: Positive; Pearson’s chi square test was used to compare proportions; *: Statistically significant at *p* < 0.05
