# Supplementary material for: Non-falciparum species and submicroscopic infections in three epidemiological malaria facets in Cameroon
Source: BMC Infect Dis. 2022 Dec 2;22:900. doi: 10.1186/s12879-022-07901-6 (PMC9718470; doi:10.1186/s12879-022-07901-6)
Supplement: Supplementary file 4 — Additional file 4. Neighbor-Joining tree of P. ovale subspecies and P.vivax in comparison to NCBI sequences based on 18S rRNA gene sequences andreference strains. [file 12879_2022_7901_MOESM4_ESM.docx]

**Additional file 4**. Neighbor-Joining tree of *P. ovale* subspecies and *P. vivax* in comparison to NCBI sequences based on 18S rRNA gene sequences and reference strains


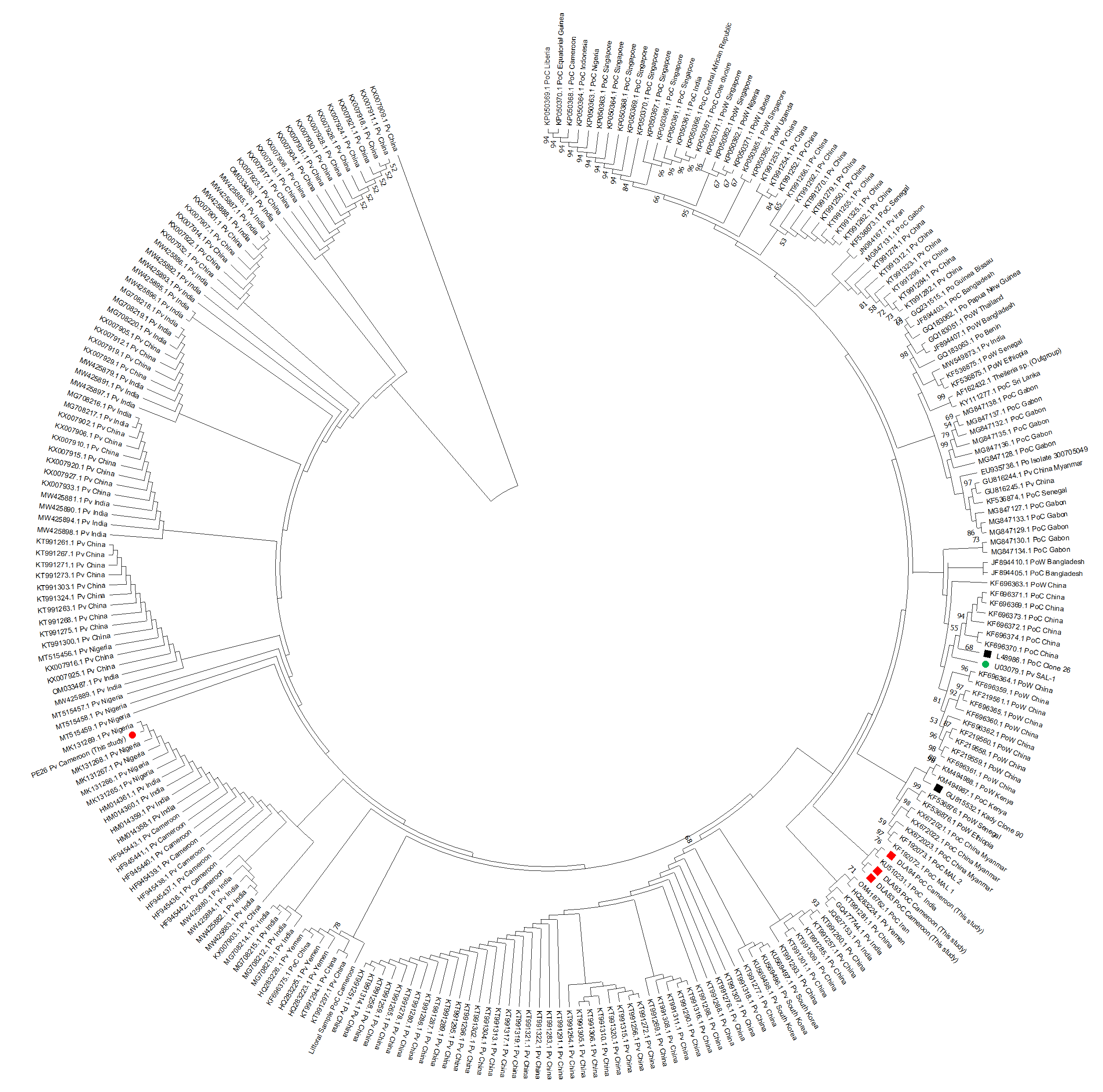


Nodal values represent bootstrap probabilities based on 1000 replicates. *PoC*: *P. ovale curtisi*, *PoW*: *P. ovale wallikeri*, *Pv*: *P. vivax*. Shapes were used to indicate *P. vivax* sample (red round shape), *P. ovale curtisi* (red square shape), reference *Pv* strain (green round shape) and reference *Po* strains (black square shape).
